# Comparison of Bioelectrical Impedance Analysis and Dual Energy X-ray Absorptiometry for Total and Segmental Bone Mineral Content with a Three-Compartment Model

**DOI:** 10.3390/ijerph17072595

**Published:** 2020-04-10

**Authors:** Yu-San Liao, Hung-Chou Li, Hsueh-Kuan Lu, Chung-Liang Lai, Yue-Sheng Wang, Kuen-Chang Hsieh

**Affiliations:** 1 Department of Diagnostic Radiology, Chang Gung Memorial Hospital, Yunlin 638, Taiwan; mm601200@gmail.com; 2 Department of Nursing, Chang Gung University of Science and Technology, Chiayi 613, Taiwan; 3 Department of Chemistry and Biochemistry, National Chung Cheng University, Chiayi 621, Taiwan; 4 Department of Diagnostic Radiology, Chang Gung Memorial Hospital, Chiayi 613, Taiwan; eagles0523@yahoo.com.tw; 5 General Education Center, National Taiwan University of Sport, Taichung 404, Taiwan; sk.lu2002@gmail.com; 6 Department of Physical Medicine and Rehabilitation, Puzi Hospital, Ministry of Health and Welfare, Chiayi 613, Taiwan; laipeter57@yahoo.com.tw; 7 Department of Occupational Therapy, Asia University, Taichung 413, Taiwan; 8 Department of Diagnostic Radiology, Chang Gung Memorial Hospital, Chiayi613, Taiwan; wangys@cgmh.org.tw; 9 Fundamental Education Center, National Chin-Yi University of Technology, Taichung 411, Taiwan

**Keywords:** BIA, bone mineral content, DXA, segment, multi-frequency, three-compartment

## Abstract

Modern bioelectrical impedance analysis (BIA) provides a wide range of body composition estimates such as fat mass (FM), lean body mass (LBM), and body water, using specific algorithms. Assuming that the fat free mass (FFM) and LBM can be accurately estimated by the 8-electrode BIA analyzer (BIA_8MF_; InBody230, Biospace), the bone mineral content (BMC) may be calculated by subtracting the LBM from the FFM estimates based on the three-compartment (3C) model. In this cross-sectional study, 239 healthy Taiwanese adults (106 male and 133 female) aged 20–45 years were recruited for BIA and dual-energy X-ray absorptiometry (DXA) measurements of the whole body and body segments, with DXA as the reference. The results showed a high correlation between BIA_8MF_ and DXA in estimating total and segmental LBM, FM and percentage body fat (r = 0.909–0.986, 0.757–0.964, and 0.837–0.936, respectively). For BMC estimates, moderate to high correlations (r = 0.425–0.829) between the two methods were noted. The percentage errors and pure errors for BMC estimates between the methods ranged from 33.9% to 93.0% and from 0.159 kg to 0.969 kg, respectively. This study validated that BIA_8MF_ can accurately assesses LBM, FM and body fat percentage (BF%). However, the estimation of segmental BMC based on the difference between FFM and LBM in body segments may not be reliable by BIA_8MF_.

## 1. Introduction

Body composition is closely related to human physiology and disease, and has a wide range of applications in nutrition, exercise physiology, medicine, geriatrics, and fitness [1,2,3,4]. The most commonly used methods of measuring body composition are hydrodensitometry, air displacement plethysmography, bioelectrical impedance analysis (BIA), and dual-energy X-ray absorptiometry (DXA) [5]. In recent years, BIA has been widely used in clinical practice, in epidemiological studies, and by individuals on a personal basis, because it is non-invasive, simple to perform, and convenient. Basically, BIA measures the electrical impedance of the water compartment to the flow of either single or multi-frequency currents. However, the human body is not a homogeneous cylinder, and body composition estimation from the impedance requires a number of assumptions and a specific regression equation for the target population [6,7]. DXA is a three-compartment model of body composition analysis [8]. During the analysis, the body is scanned with X-rays of two different levels of energy. Therefore, the DXA scanner is originally designed to separate the body into two components of known X-ray attenuation coefficients. With the application of specific soft tissue algorithms to assume the lean-to-fat fraction, the DXA scanner is able to partition the human body into the fat, lean, and bone components [9,10]. DXA, with high accuracy and precision, is commonly used as a reference method for developing and validating BIA equations [7] Redistribution of the body fluid affects body impedance and influences the accuracy of BIA measurements. It is generally agreed that BIA measurements should be taken after the subjects in the testing positions (supine or standing) for at least 10 min to avoid gravity-related shifts in body fluid [11,12]. Standing BIA devices do not require subjects to lie down on the bed prior to measurement and application of adhesive electrodes; and therefore, are more convenient and comfortable than supine BIA models.

Bone mineral contents (BMC) may change due to aging, physical training or disease processes, with varying degrees across body segments. In postmenopausal women, there is a reduction in total body bone mass, with axial bone predominance [13,14]. Athletes build bone mass in different body regions and at different rates depending on the type and intensity of their training [15,16]. It is known that the BMC of the extremity is positively correlated with muscle strength and may have an impact on athletic performance [17]. DXA is the gold standard for measuring total body and regional BMC, but has the disadvantages of radiation exposure and less availability. Similar to DXA, commercial octopolar BIA is also a body composition method providing estimates in the total body and body, enabling faster, cheaper and safer measure for body composition. Estimates of body fat percentage (BF%), fat mass (FM), fat free mass (FFM), and lean body mass (LBM) have been validated in the total body [18,19] and in body segments [20,21,22]. However, less is known about the estimation of BMC by BIA. 

BIA has been used to estimate total body BMC. However, no published research to date has investigated the application of the BIA method in measuring the limb BMC. We assumed that the BMC can be obtained after subtracting the lean and fat masses from the total body weight, based on the human three-component model, and validated the BMC measure at each limb. BIA is currently applied to measuring the muscle, fat, and water. Our study provides validation of whole-body and regional BMC estimates so that BIA can be used in general and clinical applications in the future. 

The logical of BMC calculus is that BMC is the subtraction of FFM and LBM and with the premise assumption of both FFM and LBM can be accurately measured by BIA. However, less is known about the validity and reliability of BMC measured by BIA [23]. Stone et al. [24] concluded that using single-frequency hand-to-foot BIA to measure BMC would be inappropriate for diagnostic purposes. Recently, it has been suggested that the BIA instrument does not appear to be useful in estimating BMC in healthy children [25]. This study aimed to test the accuracy and agreement of whole body and segmental BMC measurements using a portable 8-electrode multi-frequency BIA (BIA_8MF_) device using DXA as a three-compartment reference model.

## 2. Materials and Methods

### 2.1. Study Design

This cross-sectional study was approved by the Institutional Review Board of Chang Gung Memorial Hospital (103–1516B), and all participants provided their written informed consent. Participants were recruited via hospital advertisements and word of mouth between October and December 2014. The study consisted of one clinic visit. Participants were asked to fast overnight for at least 8 h before reporting to the Chang Gung Memorial Hospital (Chiayi branch). Vigorous activities and alcohol were avoided for ≥ 24 h before the study day. On arrival, participants were asked to void and change into a hospital gown. Body weight and height were measured to the nearest 0.1 kg and 0.1 cm, respectively, using a digital scale (Super-View, HW-3050, Taipei, Taiwan). The total study time was approximately 1 h. 

### 2.2. Participants

The inclusion criteria were healthy Taiwanese adults 20 to 45 years of age. Individuals with a chronic illness, diabetes, hypertension, metal implants, or who were taking any medications were not eligible for participation. Subjects were asked to abstain from vigorous exercise, alcohol, and caffeine in the 48 h before the test, and empty their bladder 30 min beforehand. Subjects were excluded if they had taken medicine that may affect hydration status in the seven days prior to the test. Female subjects were not schedule during menses. Participants are healthy individuals with normal mobility.

### 2.3. Body Composition Measurement

Body composition was assessed using a fan-beam DXA scanner equipped with software version 12.5 (Delphi A, Hologic, Bedford, MA, USA), and a dual-frequency (20 kHz and 100 kHz) BIA device (InBody230, Biospace Corp., Seoul, Korea). The test protocols for DXA and BIA_8MF_ are described elsewhere [20]. A qualified DXA technician performed all whole-body scans and analyses using the Hologic Delphi A scanner. All BIA_8MF_ measurements were made by trained research assistants. The body composition measurements provided data on LBM, FM, BF%, and BMC for the total body, trunk, right arm, left arm, right leg, and left leg. BIA_8MF_ did not directly output BMC data. In this study, BMC was the sum calculated by subtracting segments FM and segments LBM from each body segments weight. 

### 2.4. Statistical Analysis

The statistical software package SPSS version 19.0 for Windows (SPSS Inc., Chicago, IL, USA) was used for data analysis. All data were normally distributed and presented as means ± standard deviation (SD). Pearson correlation coefficient was used to measure the strength of the association between body composition measures by DXA and BIA_8MF_. The paired t-test was used to compare 2 means of measurements by DXA and BIA_8MF_. The pure error (PE) was calculated as the root mean square of the difference between observed and predicted data, as shown below:

(1) $$\mathrm{PE}=\sqrt{\frac{\sum^{\;}{\left(Predicted\;value-Observed\;value\right)}^{2}}{Number\;of\;observations}}$$

Bland-Altman analysis and plotting was used to assess the degree of agreement between DXA and BIA_8MF_ measurements. The mean paired difference (bias), and the limits of agreement (LOA, mean ± 2 SD) were calculated. The bias and LOAs in the Bland-Altman plot were unscaled values based on the original unit, and therefore the LOA% was used to present the level of precision of BIA measurements. The LOA% was calculated as shown below:

$$\mathrm{LOA}\%=\frac{Limit\;of\;agreement}{mean\;value\;by\;DXA}\times 100\%$$

The LOA% was also used to describe the level of precision of BIA measurements. Bland-Altman linear regression was used to test the relative agreement between the two methods. The statistical significant level was set at *p* < 0.05.

## 3. Results

A total of 239 participants (106 male, 133 female) were included in the study. Participant demographic data are summarized in Table 1. Male participants were significantly older (males 35.1 ± 5.8 years; females 32.7 ± 5.8 years), taller (male 172.3 ± 6.1 cm; female 161.1 ± 5.2 cm), and heavier (male 74.5 ± 11.7 kg; female 56.6 ± 8.9 kg) than female participants. The waist circumference of males was also significantly greater than that of females (male 84.9 ± 9.5 cm; female 72.9 ± 8.2 cm). The BMI of the participants ranged from 16.9–34.0 kg/m^2^.

LBM, FM, BF%, and BMC measured by DXA and BIA_8MF_ are shown in Table 2. In general, males had higher LBM and BMC, but lower BF% in the total body and segmental body levels than females. All of the body composition measurements by DXA and BIA_8MF_ were significantly different between the sexes, except for the FM, BMC, and BF% measurements in the legs of males. 

**Figure 1:**
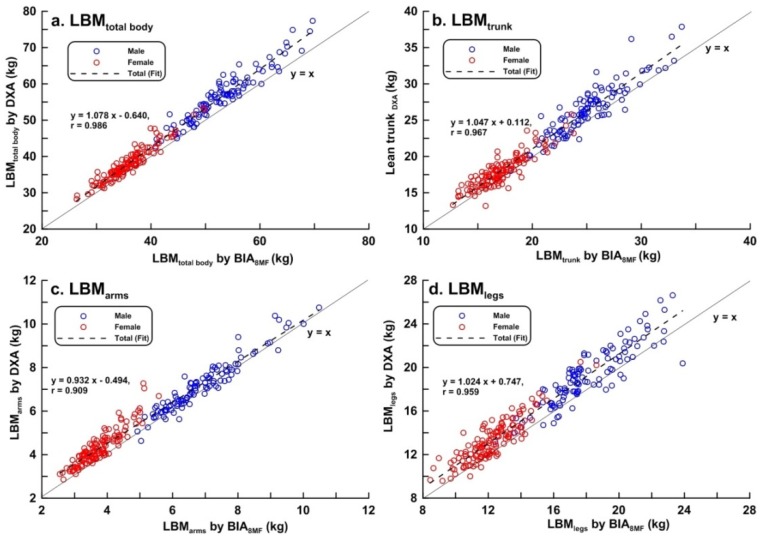
Scatter plots and linear regression line showing the relationship between dual-energy X-ray absorptiometry (DXA) and multi-frequency bioelectrical impedance analysis (BIA_8MF_) measurements of lean body mass (LBM) (n = 239). (**a**) Total body; (**b**) trunk; (**c**) arms; (**d**) legs. The bold line represents the identical line, the dotted line represents the regression line.

**Figure 2:**
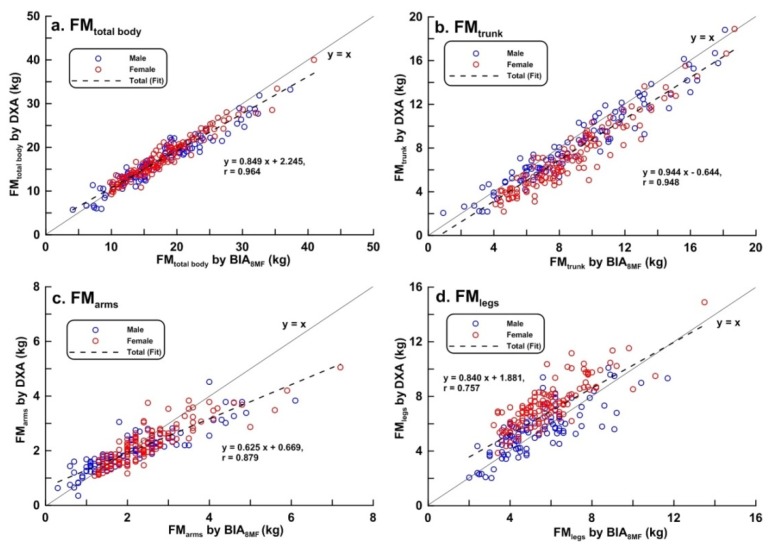
Scatter plots and linear regression line showing the relationship between dual-energy X-ray absorptiometry (DXA) and multi-frequency bioelectrical impedance analysis (BIA_8MF_) measurements of fat mass (FM) (n = 239). (**a**) Total body; (**b**) trunk; (**c**) arms; (**d**) legs. The bold line represents the identical line, the dotted line represents the regression line.

**Figure 3:**
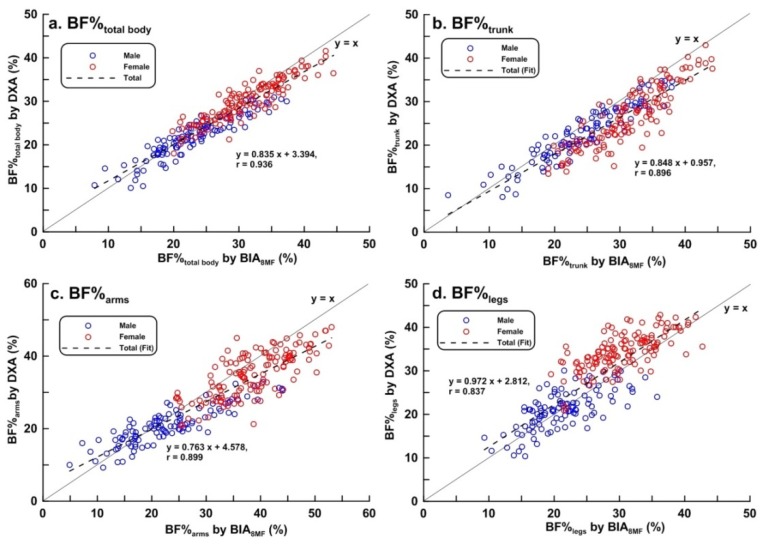
Scatter plots and linear regression line showing the relationship between dual-energy X-ray absorptiometry (DXA) and multi-frequency bioelectrical impedance analysis (BIA_8MF_) measurements of percentage body fat (BF%) (n = 239). (**a**) Total body; (**b**) trunk; (**c**) arms; (**d**) legs. The bold line represents the identical line, the dotted line represents the regression line.

**Figure 4:**
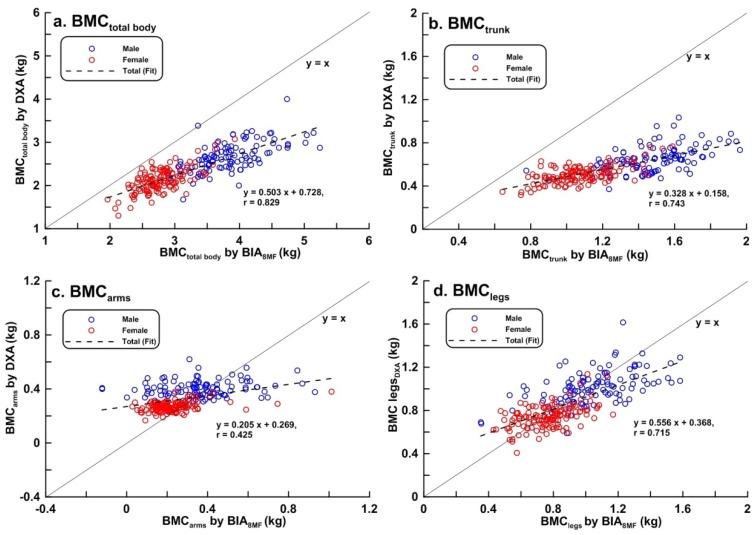
Scatter plots and linear regression line showing the relationship between dual-energy X-ray absorptiometry (DXA) and multi-frequency bioelectrical impedance analysis (BIA_8MF_) measurements of bone mineral content (BMC) (n = 239). (**a**) Total body; (**b**) trunk; (**c**) arms; (**d**) legs. The bold line represents the identical line, the dotted line represents the regression line.

Scatter plots of LBM, FM, BF%, and BMC of the total body, trunk, arms, and legs measured by BIA_8MF_ and DXA are shown in Figure 1, Figure 2, Figure 3 and Figure 4, respectively. At the total body level, the correlation coefficients for LBM, FM, BF%, and BMC were 0.986, 0.964, 0.936, and 0.829, respectively. In the trunk region, the correlation coefficients for the LBM, FM, BF%, and BMC were 0.967, 0.948, 0.896, and 0.743, respectively. In the arms, the correlation coefficients for the LBM, FM, BF%, and BMC were 0.909, 0.879, 0.899, and 0.425, respectively. In the legs, the correlation coefficients for the LBM, FM, BF%, and BMC were 0.959, 0.757, 0.837, and 0.715, respectively. In summary, the correlations between BIA and DXA measurements were the highest for LBM, and lowest for BMC, in both total body and segmental body levels. The correlations between measurements by the 2 methods were higher at the total body level than in segmental levels. It is worth noting that the correlation between measurements by the 2 methods of BMC_arms_ (r = 0.425) and BMC_legs_ (r = 0.714) were relatively lower compared to the other body composition measurements. 

Agreements between the 2 methods of the total cohort, and males and females are presented in Table 3. The LOA% for the total body and segmental measures of LBM, FM, BF%, and BMC were 8.3–29.5%, 19.5–32.8%, 18.7–32.8%, and 33.9–93.0%, respectively. The LOA% for all body composition measures was lower for the total body than segmental levels, except for BF%_trunk_ in males and BF%_arms_ in females. Among all the body composition measures, the LOA% was the lowest for LBM and highest for BMC for both sexes. Regression analysis for differences on average showed a significant change in the slope of the regression line indicating a proportional difference, except for LBM_arms_, FM_trunk_, and BF%_trunk_. The ratio of PE and the mean found by DXA was 40.6% (0.959/2.360), 125.8% (0.717/0.57), 48.2% (0.159/0.33), and 19.8% (0.173/0.87) in the total body, trunk, arms, and legs, respectively. These results indicated a limited precision of BIA_8MF_ measurement of BMC in the total body, trunk, and arms.

## 4. Discussion and Conclusions

There is increasing interest in the use of BIA as a tool for body composition assessment in research and clinical settings. BIA is regarded as a two-compartment model which divides the body into fat mass and fat-free mass. This study explored the feasibility of estimating BMC using BIA, showing that poorest correlation was noted for BMC between BIA_8MF_ and DXA among all the body composition estimates tested. When dividing the body into regions, the correlation coefficients for BMC between BIA_8MF_ and DXA was high at total body, trunk and leg levels whereas the correlation was low in the arms. Agreements between methods were also explored and showed that BMC in the whole body, and each limb may be significantly less reliable than other body composition estimates due to the limitations of the BIA methodologies.

Multi-frequency BIA is superior to single-frequency BIA, as the former can provide estimates of both intra- and extra-cellular fluid, whereas the latter can only provide an estimate of total body water. The first report on multi-frequency BIA was published by Segal et al. [26]. The superior fundamental theorem of multi-frequency segmental BIA is based on that the four limbs account for about 35% total body volume, but they contribute up to 85% of the total body impedance. Segal et al. [27] reported a moderate to strong correlation (r = 0.68 to 0.93) between total body water and whole body impedance, and a weaker correlation (r = 0.70) between extracellular fluid and whole body impedance. Subsequent validation studies on BIA_8MF_ using tracer dilution as the reference method have suggested that BIA at a frequency of 500 kHz provides the best prediction of total body water [28,29], and a frequency of 5 kHz provides the best prediction for extracellular fluid [28]. Validation studies on LBM [27,30], FM, BF% [31], and skeletal muscle mass (SMM) [32] for BIA_8MF_ have also been performed.

Using BIA to measure body composition is based on the theory that different biological tissues have different electrical properties (impedance). Therefore, the amounts of FFM and FM can be estimated using predictive equations with variables including impedance and other body indices [27,33]. Unfortunately, there is a lack of methodology for using BIA in BMC measurement. One possible solution is to calculate BMC using the difference between FFM and LBM estimated by BIA. However, while this might be feasible for prediction of BMC at the total body level, it would likely be less reliable for the estimation of BMC at segmental body level because calculation of segmental body composition involves more variables and assumptions [34]. The correlation coefficient between BIA_8MF_ and DXA measured on the BMC_total_ body is r = 0.829, which is highly correlated. However, the further analysis results of BIA_8MF_ and LOA% of Bland-Altman Plot showed that the accuracy of BIA_8MF_’s measurement results is limited. The measurement results of BIA_8MF_ on BMC_turnk_, BMC_arms_, BMC_legs_ is lower than the BMC_total_ on accuracy by the methods of correlation, regression equation or by both the Bias and LOA% of Bland-Altman Plot. The measurement results of BIA_8MF_ on BMC are also lower than other body composition items, such as LBM, FM, and BF%. At the total body level, our study showed a strong correlation between BIA_8MF_ and DXA measurements of FM and BF%, and the LOAs between the 2 methods were −6.296 kg to 7.200 kg for FM and −8.70% to 10.998% for BF%. Other studies have measured body composition with the BIA_8MF_ using DXA as the reference [19,35]. Karelis et al. [19] reported correlation coefficients of 0.97 and 0.97 for FM and BF%, respectively, and LOAs of −3.5 kg to 3.7 kg and −5.1% to 5.4% in healthy adults. von Hurst et al. [35] reported the correlation coefficient for BF% between BIA_8MF_ and DXA was 0.938 and the LOA was −4.25% to 8.37% in adults. Our study showed a similar degree of correlation, but a wider LOA. Validation studies for the total body composition estimates of other standing BIA_8MF_ models, such as InBody520, InBody720, InBody770, from the same manufacturer (Biospace Co. Ltd., Seoul, Korea) have also been published and show variable degrees of agreement [32,36].

Loss of SMM is associated with aging, disease, and development of frailty. Magnetic resonance image (MRI) is ideal for assessing SMM as it does not use ionizing radiation, and provides a direct measurement of SMM. However, MRI is limited for large scale studies due to its high cost. DXA is more suitable for large scale surveys because it has the advantages of being fast, convenient, and inexpensive. However, DXA only provides 2D data, not cross-sectional images, and therefore cannot distinguish the fat distributions in the subcutaneous, intermuscular, or intramuscular compartments. Kim et al. [37,38] showed that the appendicular LBM estimated by DXA was highly correlated with SMM estimated by whole-body MRI in adult and children. Shih et al. [39] also demonstrate a high correlation between lower limb LBM measured by DXA and lower limb SMM measured by MRI in adults. Theoretically, if the appendicular LBM by DXA can be accurately used to estimate the SMM, the limb impedance by BIA_8MF_ may be used to estimate SMM.

There have been some studies that investigated the accuracy of assessing appendicular LBM using the BIA_8MF_. Karelis et al. [19] showed that the appendicular LBM measured by the BIA_8MF_ and DXA was not correlated to poorly correlated, and with proportional bias. Lee et al. [16] reported the appendicular LBM measured with the BIA_8MF_ and DXA was highly correlated, but with proportional bias in children. Similar to their results, proportional bias was observed for LBM_arms_ and LBM_legs_ between BIA_8MF_ and DXA in our study. However, our study showed a high correlation for LBM_arms_ and LBM_legs_ between the two methods. Possible explanations for these discrepancies may be due to differences in the study participants’ race and age.

Due to the limited validation studies of the BIA_8MF_, it is difficult to conclude the degree of accuracy of BIA_8MF_ for segmental LBM measurement. However, there have been a number of validation studies of the InBody720 in a wide range of populations, such as in hemodialysis patients [40], female college athletes [21], frail older women [41], and middle-aged adults [42]. All of these studies show good precision or agreement in segmental body composition measurement between the InBody720 and DXA. Although one study showed that the InBody720 underestimated appendicular LBM and overestimated appendicular FM in old adults [42], correlations between the two methods for segmental body composition measures are high (r ≥ 0.88), suggesting the InBody720 is a good alternative for estimating segmental body composition.

In this study, the role of BIA as a three-compartment body composition method was explored. Current scientific methodologies do not support BIA as a method for measuring BMC. However, the performance of BIA in estimating BMC could be evaluated using regression analysis method with the results from a reference method. In this study, good precision and accuracy similar to previous studies was noted in the BF%, BFM and FM measures by BIA_8MF_. However, a lower degree of precision and accuracy was demonstrated in the BMC estimate. Care should be taken when interpreting BMC obtained from BIA. In BIA, the weights in the five separate body regions are obtained indirectly but not directly. Theoretically, it is possible to have greater estimation errors of BIA in the body segments than total body. Therefore, the segmental BMC obtained from BIA may be of limited value.

Several strengths and limitations of this study should be noted. The subjects recruited in the current study were healthy individuals with a narrowing age range. Additionally, the sample size was large. These may be helpful in the validation study. There are several limitations to this study. First, the age range of the participants was narrow, and thus the results may not be applicable to middle-aged and older adults. Second, the reference method used in this study was DXA, not the criterion 4-compartment model. Validation studies of body composition assessments by DXA using criterion methods have shown that the accuracy and precision is dependent on the scan modes, models, manufacturer, and subject population [43,44,45]. Third, the BMI range of the participants was 16.9–34.0 kg/m^2^, and thus the results may not be applicable to severely underweight or morbidly obese adults.

BIA results are based on prior assumptions of specific body geometry, constant body hydration level, and body water distribution [46]. Violation of the assumptions may produce errors in the analysis of body composition. Obese subjects are known to have lower water content of fat free mass, it is not surprising that BIA devices underestimate the BF% in obese subjects [18]. To avoid errors from violating BIA assumptions, this study recruited healthy adults with a mean BMI of 23.3 ± 3.6 kg/m^2^, which was about the BMI cut-off point for overweight in Asian populations.

In summary, this study found high accuracy and agreement in the LBM and FM estimates between the BIA_8MF_ (InBody230) and DXA in healthy Taiwanese young adults. However, there are limitations of the BIA_8MF_ (InBody230) for BMC measurement.

## Figures and Tables

**Table 1:** Participant physical characteristics.

	All	(n = 239)	Males	(n = 106)	Females	(n = 133)
Age (y)	33.8 ± 5.9	(20.0, 45.0)	35.1 ± 5.8	(23.0, 45.0)	32.7 ± 5.8 ***	(20.0–45.0)
Height (cm)	166.0 ± 7.9	(143.5, 189.0)	172.3 ± 6.1	(160.4, 189.0)	161.1 ± 5.2 ***	(143.5–174.8)
Body weight (kg)	64.5 ± 13.6	(40.1, 108.5)	74.5 ± 11.7	(46.8, 108.5)	56.6 ± 8.9 ***	(40.1–95.4)
Body mass index (kg/m^2^)	23.3 ± 3.6	(16.9, 34.0)	25.1 ± 3.5	(17.4, 34.0)	21.9 ± 3.0 ***	(16.9–31.7)
Waist circumference (cm)	78.2 ± 10.7	(60.0, 111.0)	84.9 ± 9.5	(65.0, 111.0)	72.9 ± 8.2 ***	(60.0–101.0)
Hip circumference (cm)	94.3 ± 6.69	(69.0, 114.0)	96.3 ± 6.6	(81.0–113.0)	92.7 ± 6.4 **	(69.0–114.0)

All values are mean ± standard deviation; minimum and maximum in parentheses. * *p* < 0.05, ** *p* < 0.01, *** *p* < 0.001 sex difference by *t*-test.

**Table 2:** Body composition measured by BIA_8MF_ and by DXA.

**LBM, FM, BF%, and BMC Measured by DXA**	**All**	**(n = 239)**	**Males**	**(n = 106)**	**Females**	**(n = 133)**
LBM_total_body_ (kg)	46.14 ± 10.80	(28.24, 43.36)	56.28 ± 7.13	(38.84, 77.36)	38.06 ± 4.64	(28.24, 53.37)
LBM_trunk_ (kg)	21.72 ± 5.10	(13.20, 37.84)	26.44 ± 3.50	(17.99, 37.84)	17.96 ± 2.23	(13.20, 25.82)
LBM_arms_ (kg)	5.12 ± 1.79	(2.53, 10.48)	6.86 ± 1.14	(4.93, 10.48)	3.72 ± 0.62	(2.55, 5.80)
LBM_legs_ (kg)	15.94 ± 3.73	(9.59, 26.64)	19.23 ± 2.65	(13.18, 26.64)	13.27 ± 2.05	(9.59, 20.51)
FM_total_body_ (kg)	17.30 ± 5.40	(5.69, 39.97)	16.93 ± 5.67	(5.69, 17.62)	17.60 ± 5.18	(9.23, 39.98)
FM_trunk_ (kg)	7.65 ± 3.34	(2.04, 18.90)	8.35 ± 3.53	(2.04, 18.82)	7.09 ± 3.08	(2.19, 18.90)
FM_arms_ (kg)	2.10 ± 0.75	(0.35, 5.05)	1.96 ± 0.74	(0.35, 4.53)	2.30 ± 1.30	(1.09, 5.05)
FM_legs_ (kg)	6.58 ± 1.93	(2.03, 14.88)	5.53 ± 1.70	(2.03, 10.07)	7.54 ± 3.23	(3.85, 14.88)
BMC_total_body_ (kg)	2.36 ± 0.41	(1.31, 4.00)	2.66 ± 0.35	(1.68, 4.00)	2.12 ± 0.28	(1.31, 3.07)
BMC_trunk_ (kg)	0.57 ± 0.12	(0.32, 1.03)	0.65 ± 0.12	(0.37, 1.03)	0.51 ± 0.08	(0.32, 0.77)
BMC_arms_ (kg)	0.33 ± 0.08	(0.17, 0.62)	0.40 ± 0.06	(0.25, 0.62)	0.27 ± 0.04	(0.17, 0.38)
BMC_legs_ (kg)	0.87 ± 0.19	(0.41, 1.62)	1.02 ± 0.15	(0.59, 1.62)	0.74 ± 0.11	(0.41, 1.14)
BF%_total_body_ (%)	26.38 ± 6.25	(10.10, 41.50)	21.83 ± 4.78	(10.10, 30.90)	30.00 ± 4.74	(18.00, 41.50)
BF%_trunk_ (%)	24.94 ± 6.85	(8.10, 43.00)	22.69 ± 6.25	(8.10, 34.70)	26.73 ± 6.79	(13.40, 43.00)
BF%_arms_ (%)	28.72 ± 9.17	(9.30, 47.95)	20.85 ± 5.16	(9.30, 32.85)	35.00 ± 6.39	(20.75, 47.95)
BF%_legs_ (%)	28.49 ± 7.89	(10.35, 42.85)	21.14 ± 4.46	(10.35, 30.00)	34.35 ± 4.29	(20.85, 42.85)
**LBM, FM, BF%, and BMC Measured by BIA_8MF_**	**All**	**(n = 239)**	**Males**	**(n = 106)**	**Females**	**(n = 133)**
LBM_total_body_ (kg)	43.36 ± 9.85 ***	(26.32, 69.66)	52.28 ± 6.35 ***	(37.32, 69.66)	35.93 ± 4.18 ***	(26.32, 29.74)
LBM_trunk_ (kg)	20.65 ± 4.72 ***	(12.70, 33.70)	25.08 ± 2.98 ***	(18.00, 33.70)	17.11 ± 2.14 ***	(12.70, 23.80)
LBM_arms_ (kg)	6.03 ± 1.74 ***	(3.08, 11.64)	7.43 ± 1.34 ***	(4.21, 11.64)	4.91 ± 1.09 ***	(3.08, 9.45)
LBM_egs_ (kg)	14.84 ± 3.50 ***	(8.44, 23.89)	18.09 ± 2.31 ***	(12.43, 23.89)	12.25 ± 1.61 ***	(8.44, 18.62)
FM_total_body_ (kg)	17.76 ± 6.11 ***	(4.10, 40.90)	17.62 ± 6.64 ***	(4.10, 37.30)	17.86 ± 5.68 *	(9.80, 40.90)
FM_trunk_ (kg)	2.29 ± 1.05 ***	(0.90, 18.70)	9.05 ± 3.71 ***	(0.90, 18.10)	8.59 ± 3.01 ***	(4.10, 18.70)
FM_arms_ (kg)	2.29 ± 1.05 ***	(0.30, 7.20)	2.09 ± 1.11 *	(0.30, 6.10)	2.46 ± 0.98 ***	(1.20, 7.20)
FM_legs_ (kg)	5.59 ± 1.73 ***	(2.00, 13.50)	5.34 ± 1.79	(2.00, 10.70)	5.79 ± 1.66 ***	(3.20, 13.50)
BMC_total_body_ (kg)	3.24 ± 0.66 ***	(1.96, 5.14)	3.82 ± 0.49 ***	(2.67, 5.14)	2.78 ± 0.33 ***	(1.96, 3.93)
BMC_trunk_ (kg)	1.26 ± 0.28 ***	(0.64, 1.96)	1.49 ± 0.21 ***	(0.78, 1.96)	1.08 ± 0.18 ***	(0.64, 1.59)
BMC_arms_ (kg)	0.28 ± 0.17 ***	(−0.12, 1.01)	0.35 ± 0.19 *	(−0.12, 0.93)	0.23 ± 0.12 ***	(0.03, 1.01)
BMC_legs_ (kg)	0.90 ± 0.24 **	(0.35, 1.58)	1.02 ± 0.15	(0.35, 1.58)	0.77 ± 0.16 *	(0.43, 1.17)
BF%_total_body_ (%)	27.53 ± 6.97 ***	(7.90, 44.50)	23.14 ± 6.03 ***	(7.90, 37.30)	31.00 ± 5.59 ***	(19.80, 44.50)
BF%_trunk_ (%)	28.30 ± 7.18 ***	(3.70, 44.20)	24.53 ± 6.87 ***	(3.70, 38.00)	31.30 ± 5.92 ***	(19.00, 44.20)
BF%_arms_ (%)	31.68 ± 10.73 ***	(4.90, 53.05)	23.09 ± 8.24 *	(4.90, 44.50)	38.52 ± 6.87 ***	(24.50, 53.05)
BF%_legs_ (%)	26.44 ± 6.73 ***	(10.40, 42.70)	21.46 ± 5.09	(10.40, 35.80)	30.41 ± 5.02 ***	(19.50, 42.70)

All values are mean ± standard deviation; minimum and maximum in parentheses. Significantly different from DXA, * *p* < 0.05, ** *p* < 0.01, *** *p* < 0.001 (*t*-test).

**Table 3:** Validity of LBM, FM, BMC, and BF% predicted from BIA_8MF_ vs. respective results from DXA compared between males and females.

	Bias ^a^ (mean ± 2 SD)	LOA% ^b^	Regression Equation ^c^	r^2^	PE ^d^
**Total (n = 239)**					
LBM_total_body_ (kg)	−2.78 ± 3.85	8.3	y = −0.092x + 1.375	0.247 ***	3.380
LBM_trunk_ (kg)	−1.07 ± 2.62	12.1	y = −0.080x + 0.631	0.089 ***	1.694
LBM_arms_ (kg)	0.91 ± 1.51	29.5	y = −0.027x + 1.056	0.003	1.178
LBM_legs_ (kg)	−1.09 ± 2.17	13.4	y = −0.063x − 0.111	0.045 ***	1.528
FM_total_body_ (kg)	0.45 ± 3.37	19.5	y = 0.125x − 1.752	0.181 ***	1.743
FM_trunk_ (kg)	1.14 ± 2.14	27.9	y = 0.000x + 1.137	0.000	1.562
FM_arms_ (kg)	0.19 ± 9.43	32.8	y = 0.355x − 0.588	0.343 ***	0.563
FM_legs_ (kg)	−2.05 ± 8.74	30.7	y = −0.123x − 0.237	0.026 *	1.624
BMC_total_body_ (kg)	0.88 ± 0.80	33.9	y = 0.505x − 0.534	0.422 ***	0.969
BMC_trunk_ (kg)	0.69 ± 0.41	71.6	y = 0.051x − 0.104	0.647 ***	0.717
BMC_arms_ (kg)	−0.05 ± 0.31	93.0	y = 0.925x − 0.327	0.425 ***	0.159
BMC_legs_ (kg)	0.03 ± 0.34	39.4	y = 0.290x − 0.226	0.116 ***	0.173
BF%_total_body_ (%)	1.15 ± 4.93	18.7	y = 0.111x − 1.849	0.086 ***	2.712
BF%_trunk_ (%)	3.36 ± 6.45	25.9	y = 0.051x + 2.003	0.010	4.652
BF%_arms_ (%)	2.96 ± 9.43	32.8	y = 0.165x + 2.042	0.116 ***	5.559
BF%_legs_ (%)	−2.05 ± 8.74	30.7	y = −0.172x + 2.699	0.076 ***	4.819
**Males (n = 109)**					
LBM_total_body_ (kg)	−3.07 ± 4.16	7.4	y = −0.117x + 2.808	0.142 ***	2.768
LBM_trunk_ (kg)	−1.36 ± 3.03	11.5	y = −0.169x + 2.998	0.124 ***	1.352
LBM_arms_ (kg)	0.57 ± 1.36	19.8	y = 0.179x − 0.711	0.099 ***	0.589
LBM_legs_ (kg)	−1.14 ± 2.46	12.8	y = −0.145x + 1.583	0.080 **	1.113
FM_total_body_ (kg)	0.69 ± 3.84	22.7	y = 0.159x − 2.060	0.256 ***	1.354
FM_trunk_ (kg)	0.70 ± 2.05	24.6	y = 0.050x + 0.259	0.031	0.825
FM_arms_ (kg)	0.13 ± 1.14	58.0	y = 0.422x − 0.724	0.447 ***	0.386
FM_legs_ (kg)	−0.20 ± 2.35	42.4	y = 0.058x − 0.514	0.006	0.789
BMC_total_body_ (kg)	1.16 ± 0.76	28.7	y = 0.412x − 0.176	0.170 ***	0.813
BMC_trunk_ (kg)	0.83 ± 0.35	53.8	y = 0.685x + 0.100	0.314 ***	0.567
BMC_arms_ (kg)	−0.05 ± 0.39	109.9	y = 1.478x − 0.604	0.658 ***	0.132
BMC_legs_ (kg)	0.04 ± 0.41	40.3	y = 0.572x − 0.588	0.233 ***	0.138
BF%_total_body_ (%)	1.35 ± 4.96	22.7	y = 0.240x − 0.407	0.264 ***	1.873
BF%_trunk_ (%)	1.84 ± 4.98	22.0	y = 0.097x − 0.471	0.064 **	2.056
BF%_arms_ (%)	2.25 ± 9.06	43.5	y = 0.490x − 8.519	0.493 ***	3.355
BF%_legs_ (%)	0.32 ± 7.75	36.7	y = 0.157x − 3.032	0.233 ***	2.578
**Females (n = 130)**					
LBM_total_body_ (kg)	−2.13 ± 3.00	7.9	y = −0.106x + 1.804	0.095 ***	1.939
LBM_trunk_ (kg)	−0.85 ± 0.35	12.0	y = −0.047x − 0.023	0.009	1.021
LBM_arms_ (kg)	1.19 ± 1.47	39.5	y = 0.638x − 1.587	0.525 ***	1.021
LBM_legs_ (kg)	−1.02 ± 1.97	14.8	y = −0.202x + 1.525	0.144 ***	1.046
FM_total_body_ (kg)	0.26 ± 2.91	16.5	y = 0.095x − 1.424	0.124 ***	1.098
FM_trunk_ (kg)	1.49 ± 1.94	27.4	y = −0.022x + 1.668	0.005	1.327
FM_arms_ (kg)	0.15 ± 2.32	100.6	y = 0.293x − 0.443	0.244 ***	0.410
FM_legs_ (kg)	−1.86 ± 6.15	80.4	y = −0.017x − 1.503	0.001	1.419
BMC_total_body_ (kg)	0.66 ± 0.49	23.2	y = 0.197x + 0.177	0.051 ***	0.526
BMC_trunk_ (kg)	0.57 ± 0.28	56.0	y = 0.884x − 0.128	0.547 ***	0.438
BMC_arms_ (kg)	−0.04 ± 0.22	81.5	y = 1.354x − 0.379	0.720 ***	0.087
BMC_legs_ (kg)	0.03 ± 0.27	36.8	y = 0.457x − 0.321	0.149 ***	0.104
BF%_total_body_ (%)	0.99 ± 4.89	16.3	y = 0.173x − 4.283	0.127 ***	1.961
BF%_trunk_ (%)	4.57 ± 6.47	24.2	y = −0.146x + 8.816	0.078 **	4.173
BF%_arms_ (%)	3.53 ± 4.80	13.7	y = 0.084x + 0.443	0.012	4.433
BF%_legs_ (%)	−3.93 ± 7.59	22.1	y = 0.186x − 9.949	0.044 *	4.071

* *p* < 0.05, ** *p* < 0.01, *** *p* < 0.001 significant correlation. Results of the LOA analysis are given as bias and 95% limits of agreement (±2 standard deviation (SD)) for LBM, FM, BMC, and BF% measured by DXA and compared with prediction by BIA. ^a^ Bias was calculated as result obtained from BIA_8MF_ measurement minus DXA. 95% Limit of agreement was calculated as ± 2 SD. ^b^ LOA% are given as a percentage of mean value measured by the DXA. ^c^ Linear regression analysis was used to determine the relative agreement between different methods. ^d^ r^2^ was calculated as determined coefficient for the relationship between (Result_DXA_ + Result_BIA8MF_)/2 and the bias.

## Abbreviations

LOA	limit of agreement;
FM	fat mass;
BF%	percentage body fat.
